# All-Solid-State Sodium-Selective Electrode with a Solid Contact of Chitosan/Prussian Blue Nanocomposite

**DOI:** 10.3390/s17112536

**Published:** 2017-11-03

**Authors:** Tanushree Ghosh, Hyun-Joong Chung, Jana Rieger

**Affiliations:** 1 Department of Chemical and Materials Engineering, Faculty of Engineering, University of Alberta, Edmonton, AB T6G 1H9, Canada; tanushre@ualberta.ca; 2 The Institute for Reconstructive Sciences in Medicine (iRSM), Misericordia Community Hospital, Edmonton, AB T5R 4H5, Canada; 3 Department of Communication Sciences and Disorders, Faculty of Rehabilitation Medicine, University of Alberta, Edmonton, AB T6G 2G4, Canada

**Keywords:** chitosan, Prussian blue, nanocomposite, sensor, chronopotentiometry

## Abstract

Conventional ion-selective electrodes with a liquid junction have the disadvantage of potential drift. All-solid-state ion-selective electrodes with solid contact in between the metal electrode and the ion-selective membrane offer high capacitance or conductance to enhance potential stability. Solution-casted chitosan/Prussian blue nanocomposite (ChPBN) was employed as the solid contact layer for an all-solid-state sodium ion-selective electrode in a potentiometric sodium ion sensor. Morphological and chemical analyses confirmed that the ChPBN is a macroporous network of chitosan that contains abundant Prussian blue nanoparticles. Situated between a screen-printed carbon electrode and a sodium-ionophore-filled polyvinylchloride ion-selective membrane, the ChPBN layer exhibited high redox capacitance and fast charge transfer capability, which significantly enhanced the performance of the sodium ion-selective electrode. A good Nernstian response with a slope of 52.4 mV/decade in the linear range from 10^−4^–1 M of NaCl was observed. The stability of the electrical potential of the new solid contact was tested by chronopotentiometry, and the capacitance of the electrode was 154 ± 4 µF. The response stability in terms of potential drift was excellent (1.3 µV/h) for 20 h of continuous measurement. The ChPBN proved to be an efficient solid contact to enhance the potential stability of the all-solid-state ion-selective electrode.

## 1. Introduction

An ion-selective electrode, whose sensing ability stems from specially-designed membranes that transduce the chemical potential of a target ion into electric potential, is a crucial component of an electrochemical ionic sensor. Most ion-selective electrodes measure the electrochemical potential difference against a reference electrode in a near zero current or in an open circuit condition [1]. Conventional ion-selective electrodes (ISE) contain liquid contacts between the metallic electrode and the ion-selective membrane. The liquid contact has several drawbacks such as changes in solution volume due to evaporation, which alters osmotic pressure [2]. The pressure and the ionic strength difference across the selective membrane drive water to transport into or out of the inner filling solution. The net water exchange results in a large volume change in the inner solution, leading to the delamination of the sensing membrane [2]. Another disadvantage of the liquid contact is a drift in the electrochemical potential. During the operation of the ISE, an interfacial charge accumulates near the membrane, and it produces a blockage in the charge transfer to the metallic surface. As a result, the output ISE potential appears to be noisy and drifting over time [3].

Solid-State Ion Selective Electrodes (SS-ISEs), where the junction between the sensing membrane and the metallic electrode is in the solid state, have been suggested as an alternative approach to overcome the limitations of the conventional liquid junction ISEs. Since the first invention in 1970 by Hirata and Date [4], SS-ISEs have been improving their output stability and reliability by introducing novel materials, often with nanostructures.

The key parameters to evaluate the electrochemical performance of SS-ISEs include stability against the potential drift, sensitivity and selectivity to the target ion. The development of novel solid contacts has been leading the improvement of the ion-to-electron transduction, the increase of transmembrane ion fluxes and the reduction of water accumulation at the electrode-membrane interphase [2]. Over the last four decades, most of the reported solid contacts have been made of conducting polymers and nanostructured inorganic materials [5,6]. In recent years, understanding the mechanism of ion-to-electron transduction has led to a remarkable performance improvement of the SS-ISEs [2,7].

Employing conducting polymers, such as polypyrrole [8], poly(3-octylthiophene) (POT) [9], polyaniline [10] and poly(3,4-ethylenedioxythiophene) (PEDOT) [11,12], has been effective in achieving high redox capacitance and electronic conductivity. The advantages of the conducting polymers include high pH sensitivity [13,14] and the surface confined ion-to-electron transduction [15], as well as high redox capacitance and electronic conductivity. However, a critical disadvantage of the conducting polymers is that the high electroactivity facilitates non-specific chemical reactions, which cause a potential drift of the SS-ISE in potentiometric sensors [13].

Carbonaceous nanostructured materials, such as three-dimensionally ordered macroporous carbon [16], carbon nanotubes [17], fullerene [18,19], graphene [20,21,22,23], colloid imprinted mesoporous (CIM) carbon [24] and porous carbon spheres [25], have also been employed as solid contacts for efficient ion-to-electron transduction. These materials are chemically stable and exhibit a large surface area with high double layer capacitance. The ion-to-electron transduction relies on the quantity of charge in the electrical double layer. In addition, electroactive redox species such as fullerene and tetrathiafulvalene (TTF) [26,27], ferrocene [28], Prussian blue [29], lipophilic Co(II)/Co(III) salts [30,31] and 7,7,8,8-tetracyanoquinodimethane (TCNQ) [32], were also used for the ionic signal transduction through the redox reaction.

Chitosan, one of the most abundant biopolymers from natural resources, is a cationic linear polysaccharide composed of randomly-distributed β-(1-4)-linked d-glucosamine (deacetylated unit) and *N*-acetyl-d-glucosamine (acetylated unit). Chitosan offers biocompatibility, biodegradability and antibacterial activity, which are suitable traits for various biomedical applications, such as drug delivery, tissue engineering and wound dressing [33,34,35]. Electrochemically, chitosan membranes possess a high double layer capacitance due to the charge distribution along the polysaccharide backbone [36]. Chitosan-based composites with metallic and carbonaceous nanoparticles have caught attention as anode or cathode materials in sodium ion battery research [36]. Chitosan has been used in supercapacitors as a capacity enhancer or as a binder material for electrodes [37,38,39,40] and also as a host material for aqueous and non-aqueous gel for electrolytes [41]. The capacitive nature of chitosan has been improved by nitrogen doping [42,43]. The addition of graphene is known to improve conductivity [44,45]. The versatility of chitosan-based membranes in electrochemical devices motivates their application in the SS-ISE as the solid contact for potentiometric ion-selective sensors.

The chitosan-based membrane can be employed as the solid contact in the SS-ISE for ion sensing applications when the redox activity is improved and ion-specific transducing ability is included. Prussian blue (PB), or ferric hexacyanoferrate, has a face-centered cubic structure, the lattice of which consists of alternating iron(II) and iron(III) ions. The structure allows size-specific entrapment of alkali ions that can allow efficient redox interaction for potentiometric sensor applications [46,47]. Sodium ion insertion (sodiation) and extraction (desodiation) in and out of the PB nanoparticles were utilized as the cathode material for sodium ion batteries [48,49]. The combination of chitosan and PB has been employed in various applications such as drug delivery, gene delivery, amperometric biosensors, immunosensors, fuel cells and batteries [50,51,52]. The chemical and electrochemical properties of chitosan and PB and their combinatorial effect in many electrochemical applications provide enough logic to pick the materials to use as the solid contact in potentiometric ion sensors.

In the present work, we employed the chitosan/PB nanocomposite (ChPBN) as the solid contact in the solid-state sodium-sensing electrode (SS-Na^+^ISE). Here, a thin film of ChPBN was synthesized on a screen-printed carbon electrode, followed by the deposition of a sodium ion-selective membrane (Na^+^ISM). The capacitive nature of chitosan in combination with the high redox activity of PB contributed to achieving the stable potential response of the SS-Na^+^ISE. The resulting sodium selective electrode showed a near-Nernstian linear response for large concentration range (52.4 mV/decade; from 10^−4^–1 M) with remarkable potential stability.

## 2. Materials and Methods

### 2.1. Reagents and Instruments

Chitosan (low molecular weight), potassium ferricyanide (K_3_[Fe(CN)]_6_), ferrous chloride (FeCl_2_), sodium chloride (NaCl), potassium chloride (KCl), tetrahydrofuran (THF), bis[(12-crown-4)methyl]dodecylmethylmalonate (sodium Ionophore VI; Selectophore grade), 2-nitrophenyl octyl ether (2-NPOE), sodium tetraphenylborate (NaTPB) and polyvinyl butyral (PBV, Butvar^®^ B-98) were purchased from Sigma-Aldrich (Darmstadt, Germany). Acetic acid (Glacial, 98%), acetone, glutaraldehyde, Na_2_HPO_4_, NaH_2_PO_4_, NH_4_Cl, NH_4_OH and potassium phosphate buffer (50 mM, pH 7.4) were purchased from Fisher Scientific (Waltham, MA, USA). Poly(vinyl chloride) (PVC; Selectophore grade, high molecular weight) was purchased from Fluka AG (Sigma-Aldrich, Darnstadt, Germany). Phosphate buffer solution (NaPB; pH 7.4) was prepared using its monobasic and dibasic salts following the Cold Spring Harbor Protocol [53]. The 100 mM sodium phosphate buffer (NaPB; pH 7.4) was prepared by mixing 22.6 mL of 1 M NaH_2_PO_4_ (monobasic) and 77.4 mL of 1 M Na_2_HPO_4_ (dibasic) stock solutions. Then, 10 mM NaPB was prepared from 10× dilution of 100 mM NaPB. The sodium solutions were prepared in Milli-Q water, and the ionic strength adjuster (ISA) was added to each solution. The ISA stock solution was separately prepared in the laboratory with 1 M NH_4_Cl/NH_4_OH and mixed with the sodium solution to a final concentration of 80 mM. All the chemicals were of analytical grade and used as received without further purification. All the analytical solutions were prepared in Milli-Q deionized water (18.2 MΩ) unless otherwise noted. All the experiments were conducted at laboratory room temperature (20 °C).

The morphology of ChPBN was observed by transmission electron microscopy (TEM, Philips/FEI, Morgagni) operational at 100 kV. Scanning electron microscopy (SEM Zeiss EVO, Jena, Germany) and energy-dispersive X-ray (EDX, Oxford Instruments, Oxford, UK) microanalysis of cross-linked ChPBN solid contact film were performed by Zeiss Sigma field-emission SEM (Oberkochen, Germany). The Fourier-transform infrared (FTIR) spectra were recorded using the ATR-FTIR Nicolet iS50 FTIR Spectrometer (Thermo Scientific™, Waltham, MA, USA). X-ray photoelectron spectroscopy (XPS) measurements were carried out with an XPS Spectrometer (AXIS 165, Kratos Analytical, Manchester, UK). X-ray photoelectronic spectra (XPS) were obtained by using monochromatic Al Ka radiation (150 W, 15 kV, 1486.6 eV). Electrochemical characterization was carried out with the electrochemical workstation AUTOLAB potentiostat/galvanostat (PGSTAT302N, Metrohm Autolab B.V., Utrecht, The Netherlands). Screen-printed electrodes (DRP 150, DropSense, S.L., Asturias, Spain) containing the carbon working electrode, the platinum counter electrode and the Ag/AgCl (solid state) reference electrode were used to prepare the SS-Na^+^ISE. Electrochemical properties were evaluated by cyclic voltammetry (CV), chronopotentiometry (CP) and electrochemical impedance spectroscopy (EIS) modes. All the CP experiments for ISE were recorded at zero current, whereas polarization experiments were performed at fixed applied current (±1 nA and ±100 nA) for 60 s for the anodic and cathodic cycle.

### 2.2. Synthesis of Chitosan-Prussian Blue Nanocomposite

We adopted and modified the protocols reported by Zhang et al. for the synthesis of ChPBN [54]. In brief, chitosan powder was freshly dissolved in 2% acetic acid solution in deionized water and stirred at 80 °C for 3 h. The undissolved portion was removed by a 0.45-µm cellulose filter paper to obtain a homogeneous chitosan solution with a concentration of 1 mg·mL^−1^. The aqueous solution of K_3_Fe(CN)_6_ was added to the chitosan solution to the final concentration of 1 mM under magnetic stirring at room temperature. An aqueous solution of FeCl_2_ (1 mM) was then slowly added into the mixture. The mixture was stirred vigorously for 3 h. The solution mixture showed a gradual color transition from green to blue and then, finally, to dark blue, which indicates the formation of the PB. Then, the ChPBN was selectively precipitated by adding a large amount of acetone (5:1 volume ratio for acetone to the solution mixture). Then, a vigorous vortex mixing for 60 s removed the KCl impurity from the ChPBN. The KCl-removed precipitate underwent centrifugation and acetone washing three times for purification. The purified ChPBN was drop casted on the working electrode and air-dried. Finally, the ChPBN film was cross-linked with 1% glutaraldehyde (in distilled water). For comparison, the PB nanoparticle without the chitosan matrix was also prepared following the recipe in [55].

### 2.3. Preparation of Solid State-Na^+^ Ion-Selective Electrode 

**Figure 1:**
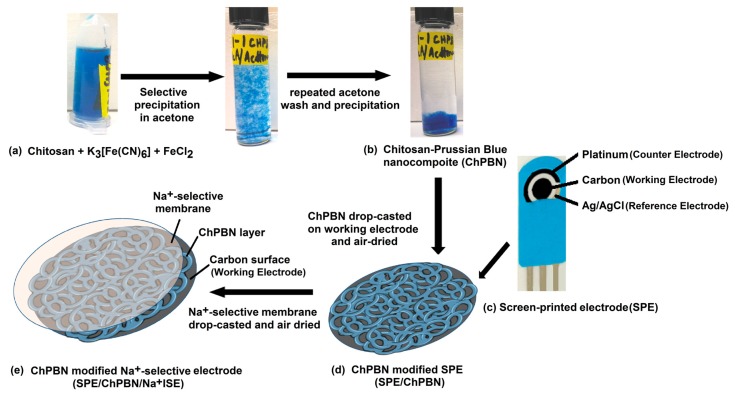
Schematics that represent the preparation of the all-solid-state sodium ion-sensing electrode (SPE/ChPBN/Na^+^ISE): (**a**) Preparation of chitosan/Prussian blue nanocomposite (ChPBN). The procedure is described in Section 2.2; (**b**) As-received carbon working electrode of the commercial screen-printed electrode (SPE); (**c**) The ChPBN solution is drop casted on the SPE then air-dried to fabricate SPE/ChPBN; (**d**) The Na^+^ ion-selective membrane (ISE) solution is drop-casted on the SPE/ChPBN and then air-dried to fabricate SPE/ChPBN/Na^+^ISE (**e**), the final ChPBN modified electrode.

The carbon working electrode (4 mm in diameter) in the as-purchased screen-printed electrode (SPE) was coated with the ChPBN membrane, followed by the formation of the sodium ion-selective membrane. These membranes were deposited by solution drop casting, followed by air-dry. The deposition of the two membranes generates SS-Na^+^ISE with the structure of SPE/ChPBN/Na^+^ISE. The cocktail solution to cast the sodium ion-selective membrane was prepared by mixing PVC (33 mg), 2-NPOE (65.45 mg) and sodium tetraphenylborate (0.55 mg) with 1 mL of THF. The cocktail solution was thoroughly mixed until it became transparent and then stored in a refrigerator at 4 °C [23]. At each time of the sodium-selective membrane coating onto the ChPBN-coated electrode, sodium ionophore VI was added (1 mg/mL) into the membrane cocktail solution to ensure the freshness of the ionophore. Air-drying of the sodium ion-selective membrane concludes the preparation of the SPE/ChPBN/Na^+^ISE. Figure 1 schematically illustrates the preparation procedure. The thickness and uniformity of the drop-casted ChPBN and PVC-based film were observed using a two-dimensional surface topography profiler (Alpha-Step IQ, KLA-Tencor, Milpitas, CA, USA). The thickness of ChPBN film was observed to be 3 µm, and the PVC-based ion-selective membrane was 64 µm. The solution for the PVB reference electrode was prepared by dissolving 79.1 mg PVB and 50 mg of NaCl into 1 mL of methanol [56,57]. The Ag/AgCl solid-state reference electrode was prepared by drop-casting 2 µL of PVB reference solution on the Ag/AgCl electrode of SPE-150.

## 3. Results and Discussion

### 3.1. Characterization of ChPBN

**Figure 2:**
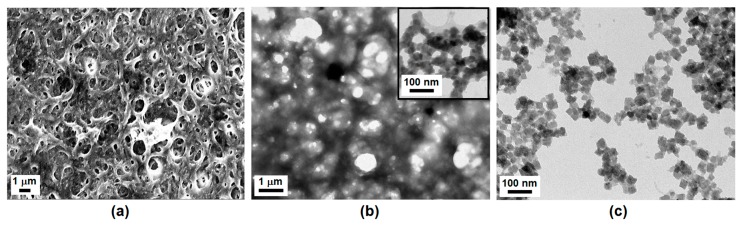
Morphological study of the chitosan/PB nanocomposite (ChPBN) film as prepared for the SS-Na^+^ISE and the control set Prussian blue (PB) nanoparticles: (**a**) SEM image of the ChPBN thin film; (**b**) TEM images of ChPBN thin film at low (main) and high (inset) magnifications; (**c**) TEM images of PB nanoparticles without the chitosan matrix.

Morphological studies using SEM and TEM provided visual information about the size, shape and the distribution of PB nanoparticles within the chitosan matrix. The SEM image in Figure 2a shows the morphology of the ChPBN thin film. Here, the SEM image clearly shows the tubular network structure of ChPBN. The tubular structure also allows the formation of pores of different magnitudes. The TEM image in Figure 2b shows the drop-casted ChPBN. The TEM image also confirmed the network structure that allows porosity. During the preparation of the nanocomposite, acidic chitosan solution and ferricyanide solution were mixed. Due to the electrostatic attraction between cationic chitosan chains and anionic ferricyanide ions, the PB nanocube formation took place at pre-adhered [Fe(CN)_6_]^−3^ sites on the chitosan polymer networks. Considering that the surface energy of PB nanoparticles is typically higher than that of polymers, it is reasonable to assume that the chitosan matrix covers the PB nanoparticles in the inset of Figure 2b. Therefore, ChPBN shows a highly porous network structure that allows the penetration of the PVC-based ion-selective membrane, which is the outer layer of the SS-Na^+^ISE. The inter-penetration of the two layers contributes to the high sensitivity and the stability of the potential response. Figure 2c shows the TEM image of PB nanocubes prepared without chitosan matrix. The sizes of the nanocubes were 40 ± 5 nm. Morphological studies notably conclude that there is the entrapment of the PB nanoparticle and thus the formation of a mesoporous composite with chitosan. 

**Figure 3:**
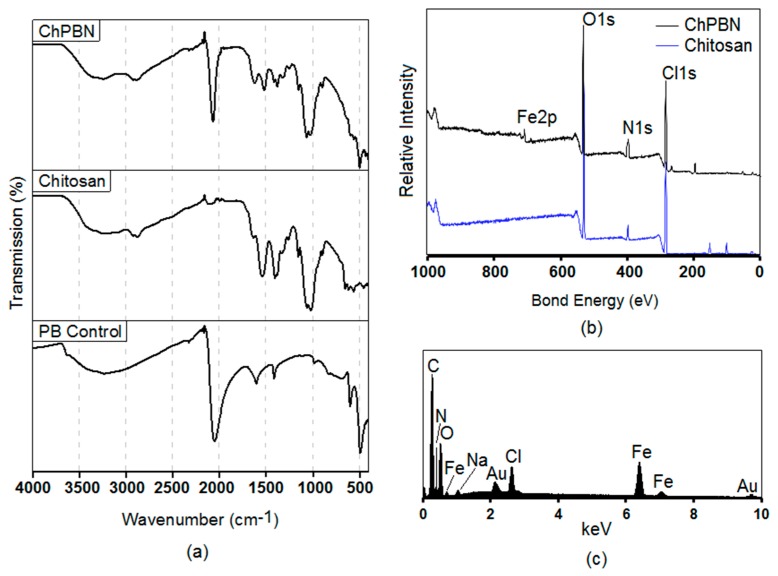
Chemical analysis of ChPBN nanocomposite: (**a**) FTIR spectra for ChPBN in comparison to chitosan and PB; (**b**) XPS spectra of ChPBN compared with chitosan showing the characteristic Fe 2p peaks indicating PB incorporation within the chitosan matrix; (**c**) EDS spectrum of ChPBN.

Figure 3a shows the FTIR spectra of the ChPBN, as well as chitosan (control) and PB (control). The PB (control) shows a strong and sharp peak for the stretching vibration of C≡N group at 2050 cm^−1^. The two peaks at 697 and 589 cm^−1^ are assigned to the stretching vibration of Fe–(C≡N) and Fe–C. All these peaks are the signature of metal hexacyanoferrates. In the FTIR spectrum of the chitosan sample, the strong peaks at 3246 cm^−1^ and at 2887 cm^−1^ of the chitosan (control) correspond to O–H and C–H stretching vibrations, respectively. The band at 1617 cm^−1^ is attributed to N–H bonds from primary amines. The low-intensity peaks at 1411 cm^−1^ and 1062 cm^−1^ correspond to the bending of the C–H bonds of the methyl groups and the stretching vibrations of the C=O bonds, respectively. All the peaks are in accordance with [58]. The FTIR spectrum of ChPBN shows signature peaks of both PB and chitosan, indicating the successful synthesis of the nanocomposite. For ChPBN, there was a significant peak around 1657 cm^−1^ corresponding to the formation of an imine bond (C=N). This is Schiff’s base structure formed by the reaction between the amino groups of chitosan and the aldehyde groups of glutaraldehyde (the cross-linking agent) [59]. The cross-linking actually occupies the amine groups of chitosan making the composite less susceptible to water intake. Only 2% swelling was observed for the thin film of ChPBN after 30 h of complete submergence in water and no further swelling was observed after seven days in water. The XPS spectra (Figure 3b) of ChPBN and chitosan (control) films show that the main peaks of N1s, O1s and C1s exist for both samples, whereas the Fe 2p peak appears only in ChPBN. A detailed analysis of the Fe 2p area revealed two peaks with lower bond energy at 706.7 eV (Fe 2p_3/2_) and 719.5 eV (Fe 2p_1/2_). These two peaks correspond to the oxidation states of Fe(II) and Fe(III), respectively. The chemical formula of the insoluble form of PB can be assumed as Fe_4_^III^[Fe^II^(CN)_6_]_3_ [60]. Figure 3c shows The EDS spectrum from ChPBN film. The EDS elemental analysis confirms the presence of Fe, C and N elemental signatures. The absence of the K elemental signal proves the successful removal of excess potassium from the ChPBN. The signature peaks for Au represent the gold surface coating for SEM imaging. All the above morphological and chemical characterization favors the claim of the successful synthesis of the nanocomposite.

### 3.2. Electrochemical Characterization of Electrodes with ChPBN Solid Contact

**Figure 4:**
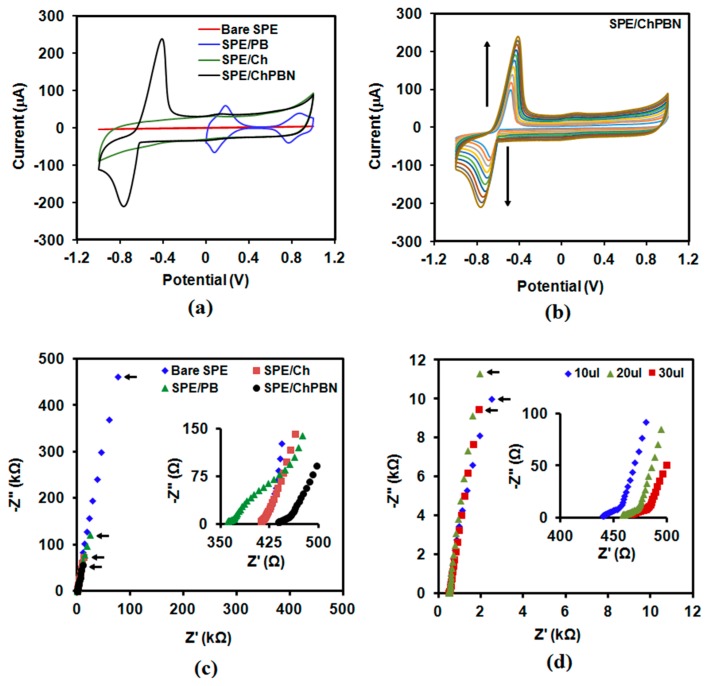
Electrochemical analysis with ChPBN thin films: (**a**) CV curves of SPE/ChPBN compared to three control samples (bare SPE, SPE/PB and SPE/Ch; see the main text for the definitions); (**b**) the effect of scan rate on the CV curves of SPE/ChPBN. Arrows indicate the increase of scan rate from 10–100 mV/s; (**c**) Nyquist plots from EIS spectra of SPE/ChPBN and the three reference samples; (**d**) the effect of droplet solution volume on the Nyquist plots of SPE/ChPBN electrodes. Insets in (**c**) and (**d**) are the magnified views of the high frequency regions. The arrows are pointing to the impedance values measured from the lowest frequency, 100 mHz, for each sample; these are the values used to extract capacitance in Table 1.

Cyclic voltammetry (CV) and electrochemical impedance spectroscopy (EIS) elucidated the electrochemical properties of the electrodes that employ ChPBN as the solid contact. CV of the SPE/ChPBN sample in the 0.1 M NaCl solution within a potential ranging from −1 V–+1 V at the scan rate of 100 mV/s revealed the redox-capacitive behavior of the nanocomposite layer. Control samples include bare SPE, the SPE coated with PB nanoparticles without chitosan (SPE/PB) and the SPE coated with a neat chitosan layer (SPE/Ch) (Figure 4a). The voltammogram of SPE/Ch did not have any redox peak, whereas SPE/PB showed redox couples at 141 mV and 826 mV. These two redox couples correspond to the conversion from Prussian white to Prussian blue and from Prussian blue to Berlin green, respectively (with increasing voltage) [61]. For SPE/ChPBN, a shift in redox peaks towards negative potential (−567 mV; from Prussian white to Prussian blue) was observed with a prominent peak separation for reduction and oxidation compared to SPE/PB. In addition, very faint redox peaks for Prussian blue and Berlin Green conversion were observed in the experimental condition. Here, the chitosan layer on PB can cause the unequal distribution of ions compared to the case that all PB nanoparticles are exposed to the solution [62]. The chitosan polymer network and PB nanoparticles acted as a cationic matrix and anionic sites in ChPBN, respectively. The high capacitive current of chitosan eventually masked the redox peaks of Prussian blue and Berlin Green conversion of PB. The generation of Donnan potential in the ChPBN can explain this phenomenon. Figure 4a shows that the capacitive current of the bare electrode is negligible. SPE/ChPBN has higher capacitive current than SPE/PB. The current outputs of SPE/ChPBN and SPE/Ch are very similar in the capacitive region (the flat regions between −0.2 V and 1 V). These indicate that chitosan contributes to the amount of capacitive charge in the nanocomposite film. The scan rate modulation was performed to predict the electron transfer mechanism for the modified electrode. Figure 4b shows the effect of scan rate on the CV characteristics of the SPE/ChPBN electrode. Here, the anodic (*Ipa*) and cathodic (*Ipc*) peak currents increased with increasing scan rate. The anodic peaks shifted by +87.6 mV when the scan rate increased from 10–100 mV/s towards positive potential. The increase in the current response with the increasing scan rate represents the reversible redox behavior of ChPBN composite film. It indicates that the chitosan coating is not affecting the electron transfer or no such chemical reaction is associated with the redox couple of PB. The plots of the Ip vs. v^1/2^ (Figure S1; Supplementary Materials) for both the anodic and cathodic half showed linearity with scan rate. This indicates fast electron transfer at the ChPBN-electrode interface. The solid contact coating on the electrode is not hindering the electron transfer mechanism in any case. The EIS studies were performed to characterize the resistance and capacitance contribution of ChPBN towards the modified electrode. Figure 4c shows Nyquist plots from the EIS spectrum (complex plane plots of −*Z*” vs. *Z*’) in 0.1 M NaCl solution at scanning frequencies from 100 kHz–100 mHz with a modulation amplitude of 0.01 V. The fitting based on the equivalent circuit models (NOVA 2.0.2 software; Metrohm Autolab B.V. Utrecht, The Netherlands) was used to extract capacitance and interface resistance values. Here, the impedance spectra for bare SPE, SPE/PB, SPE/CH and SPE/ChPBN are following a nearly vertical (90°) line at lower frequencies (capacitive line). The absence of the high frequency semicircle (the inset of Figure 4c) indicates very fast ion and electron transfers at the ChPBN/solution interfaces [11]. Following Boback’s protocol [11], we assumed the electrochemical electrode as an equivalent circuit with the interfacial resistance and the capacitance connected as a series circuit. The capacitance (*C*) can be determined from the *Z*” value of the imaginary part of impedance for the lowest frequency using Formula (1):

(1) $$C=-\frac{1}{2\pi fZ\prime \prime }$$

where *f* is frequency and *Z*” is the value of the imaginary part of impedance. Table 1 represents the capacitance values determined from the four electrodes. The effect of film thickness on the capacitance values of the SPE/ChPBN electrodes was also studied and represented in the Nyquist plots in Figure 4d. Here, the loading volume to make the drop-casted electrodes varied; larger loading generated thicker film. After applying Boback’s protocol, the capacitance values decreased about 20 ± 2 µF when the loading increased from 10–30 μL. The variance in the capacitance corresponds to 13% of the droplet capacitance from the 10 µL sample. Here, we used 10 µL of loading volume in the remainder of the article.

**Figure 5:**
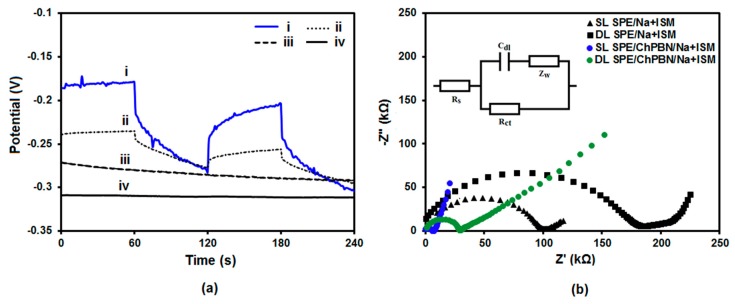
Potentiometric and impedimetric response of electrodes with and without ChPBN: (**a**) Anodic and cathodic chronopotentiometry plots for SPE/Na^+^ISE (Line i) and SPE/ChPBN/Na^+^ISE (Line ii) in 0.1 M NaCl at the periodically-alternating current of ±100 nA. Chronopotentiometry polarization at ±1 nA applied current before (Line iii) and after (Line iv) conditioning of SPE/ChPBN/Na^+^ISE in 0.1 M NaCl for 1 h; (**b**) Nyquist plots from EIS measurement of SPE/Na^+^ISE and SPE/ChPBN/Na^+^ISE in 0.1 M NaCl. SL and DL represent single layer and double layer of ChPBN, respectively. Inset: the Randles circuit proposed to fit the EIS experimental result using NOVA software (version 2.0.2; Metrohm Autolab B.V., Utrecht, The Netherlands).

Electrochemical behaviour was studied to explore the capacitance and resistance of the ChPBN solid contact of SPE/ChPBN/Na^+^ISE. The efficiency of charge exchange was evaluated by chronopotentiometry (CP) and EIS measurements. CV could not be used due to the high resistance of the PVC-based ion-selective membrane. CP was performed by consecutive application of anodic and cathodic currents (Figure 5a). For both anodic and cathodic polarization, flipping caused the instant potential jumps, followed by temporary potential drift. From the slope of the linear part of the potential drift (immediately after each potential jump), the redox capacitance can be estimated by:

(2) $$\frac{\Delta \;E}{\Delta \;t}=\frac{I}{C}$$

where *I* is applied current and *C* is capacitance [10]. It is notable that near zero currents (±1 nA) were too small to monitor polarization effects. Here, CP was performed at relatively high current (±100 nA) for 60 s of anodic and cathodic cycles to trigger potential jumps. Curves i and ii in Figure 5a compare the potential jumps and drifts for the SPE/Na^+^ISE and SPE/ChPBN/Na^+^ISE electrodes during the periodic polarization cycles. Considering that the bulk resistance of the PVC-based membrane dominates the total resistance of the electrode, the membrane resistance (*R_m_*) can be estimated by:

(3) $$R_{m}=\frac{E}{I}$$

where *E* represent the amount of the instant potential change due to the applied current *I* [10]. According to Equation (3), the membrane resistance was 372.31 kΩ for SPE/Na^+^ISE and 89.41 kΩ for SPE/ChPBN/Na^+^ISE. The amount of potential jumps and ohmic drops decreased when the ChPBN layer is incorporated in the ISE. In addition, the effect of conditioning the SPE/ChPBN/Na^+^ISE electrode in the 0.1 M NaCl solution for 1 h is shown in Curves iii (before) and iv (after conditioning) of Figure 5a. Here, alternating near zero currents (±1 nA) were applied with a 60-s interval, whereas the current could not cause potential drop. The conditioning clearly improved the potential stability.

EIS spectra were also recorded for Na^+^ISE with and without the ChPBN solid contact for a frequency range from 1 mHz–1 mHz (in 0.1 M NaCl solution; 0.01 V modulation amplitude). Figure 5b shows that all EIS spectra have semi-circular high frequency patterns and linear low frequency output. Here, the broadening of the semicircles in the Nyquist plots reflects the increase in resistance by double layer depositions. The higher resistance hinders efficient charge transfer. More explicitly, the SPE/ChPBN/Na^+^ISE electrode with the solid contact can be represented by an equivalent circuit model. The experimental data fit into a simple Randles circuit to extract the components [63]. The Randles circuit (Figure 5b, inset) represents the solution resistance (*R_s_*), charge transfer resistance (*R_ct_*) at the membrane/solution interface, the double layer capacitance (*C_dl_*) and the finite-length Warburg diffusion impedance (*Z_w_*). Table 2 summarizes the estimated potential drift (ΔE/Δt) and capacitance (*C*) values from SPE/Na^+^ISE and SPE/ChPBN/Na^+^ISE under various operating conditions. The capacity of the ChPBN layer was calculated to be 154.5 µF, which in turn contributed to the increased capacitance from 457 µF (SPE/Na^+^ISE) to 737 µF (SPE/ChPBN/Na^+^ISE). The excess capacity may come from the PVC-based membrane of Na^+^ISE. The capacitance values are comparable to conducting polymer solid contacts, such as PEDOT(PSS) of 162 µF [12], whereas the value is higher than graphene, carbon black, fullerene and carbon nanotubes. The calculated capacity values are better than those measured for TCNQ of 154 µF [32], carbon black of 51 µF [64], SWCNT of 59 µF [17], graphene sheet of 91 µF [22] solid contact electrodes. The high capacitive nature of ChPBN provides long-term potential stability and thus makes it suitable as a solid contact for all-solid-state ion-selective electrodes. Table S1 in the Supporting Information summarizes extracted parameters from 21 different systems from previous literature, clearly showing the comparison.

### 3.3. Potential Response and Stability of SPE/ChPBN/Na^+^ISE

**Figure 6:**
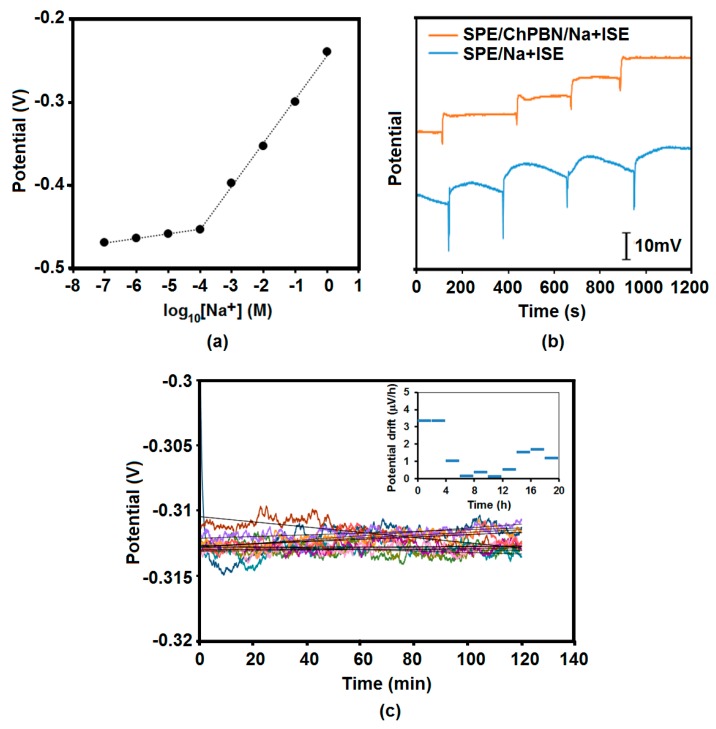
Calibration curve and potential stability study of SPE/ChPBN/Na^+^ISE: (**a**) The variation of open circuit potentials of SPE/ChPBN/Na^+^ISE with respect to [Na^+^] ranging from 10^−7^–1 M. Here, a near Nernstian slope (52.4 mV/decade) was obtained in the concentration range between 10^−4^ and 1 M; (**b**) Zero current potentiometry of SPE/Na^+^ISE and SPE/ChPBN/Na^+^ISE with respect to time. Here, the initial sodium ion concentration was 0.1 M, and each step represents the increment of 0.1 M up to 0.5 M. The comparison between the two plots shows that the addition of the ChPBN layer significantly enhanced the potential stability; (**c**) Potential stability test for SPE/ChPBN/Na^+^ISE showing ten consecutive measurements for two hours. Inset: calculated potential drift for every 2 h as a function of time.

The solid-state sodium selective electrodes (SPE/ChPBN/Na^+^ISE) were tested for Na^+^-selective response. Figure 6a shows the electrode potential of SPE/ChPBN/Na^+^ISE with respect to [Na^+^] ranging from 10^−7^ M–1 M. The sodium solutions were supplemented with 80 mM ionic strength adjuster (ISA) solution. The slope of the fitting to the linear range (10^−4^–1 M) was 52.4 ± 0.4 mV/decade. The ChPBN layer was effective in achieving stable potential, as evidenced by the time-dependent measurement in Figure 6b. Here, the output potential significantly stabilized for SPE/ChPBN/Na^+^ISE when compared to that of SPE/Na^+^ISE. The potential stability was also monitored at a fixed primary ion concentration ([Na^+^] = 130 mM) for 20 h (10 cycles of 2-h measurements; no interval between the cycles). Figure 6c shows the drift of the output potential of the SS-Na^+^ISE (SPE/ChPBN/Na^+^ISE). The potential drift of SPE/ChPBN/Na^+^ISE was 3.3 µV/h over the first 4 h (inset), whereas the drift in the later time was noticeably lower. The average potential drift over 20 h was 1.3 µV/h (3.61 × 10^−4^ µV/s), which is better than the potential drift reported for the solid contact electrodes based on porous carbon of 212 µV/s [23], graphene sheet of 55 µV/s [22], reduced graphene oxide of 12.8 µV/s [20], SWCNT of 85 µV/s [17] and TCNQ of 9.2−11.1 µV/s [32]. The reduced potential drift for ChPBN is very much comparable to polypyrrole film of 9.23 × 10^−3^ µV/s [8], polyaniline doped-POT of 9.23 × 10^−3^ µV/s [10], three-dimensionally-ordered macroporous (3DOM) carbon of 3.25 × 10^−3^ µV/s [16], colloid-imprinted mesoporous (CIM) carbon of 3.61 × 10^−4^ µV/s [24] and carbon black 4.19 × 10^−3^ µV/s [65] (see Table S1 in the Supplementary Materials). The enhanced potential stability also supports the ChPBN as a suitable material as a solid contact in ISEs.

**Figure 7:**
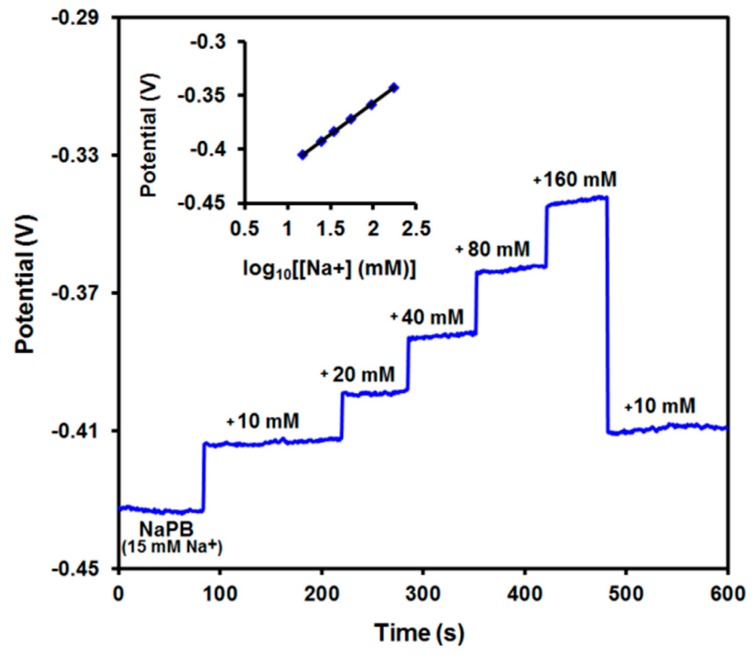
Potential responses of SPE/ChPBN/Na^+^ISE to increasing concentrations of sodium in the PBS solution. The inset shows a Nernstian slope of 58 mV/decade.

The [Na^+^] detectability of SPE/ChPBN/Na^+^ISE in the presence of background ions was tested in sodium phosphate buffer (NaPB, 10 mM, pH 7.4) with added NaCl with the concentration 10–160 mM and potential response measured at zero current. Figure 7 shows the performance of SPE/ChPBN/Na^+^ISE in sodium phosphate buffer. Here, we could confirm the near-Nernstian slope of 58 mV/(log_10_[Na^+^]). The standard curve in the Figure 6a shows the current response of the sensor only against the known concentration of added NaCl. The electrolyte background was adjusted with ammonium chloride/ammonium hydroxide ion strength adjuster solution. Therefore, the responses were detected only from Na ion activity. Furthermore, in Figure 6a, the standard curve showed two different slopes within a broad range of NaCl from 10^−7^–1 M. The lower concentration range (10^−7^–10^−4^ M) showed low potential difference with a lower slope value (sub-Nernstian response). For the higher concentration range (10^−4^–1 M), the sensor showed a near-Nernstian slope. In Figure 7, the current response was more prominent with a single slope of 58 mV. Here, the responses were measured in the presence of 10 mM sodium phosphate buffer as a background electrolyte. The NaPB of 10 mM strength actually retains 15 mM of Na^+^ ions (10 mM Na^+^ from dibasic and 5 mM Na^+^ from monobasic salts of sodium), which is additionally calculated for each supplemented solution. Each supplemented sodium concentration (10–160 mM) had added sodium ions from the background buffer. The inset of Figure 7 shows the actual measured values with the calculated sodium ion concentration.

### 3.4. Water Layer Test and Selectivity Test

**Figure 8:**
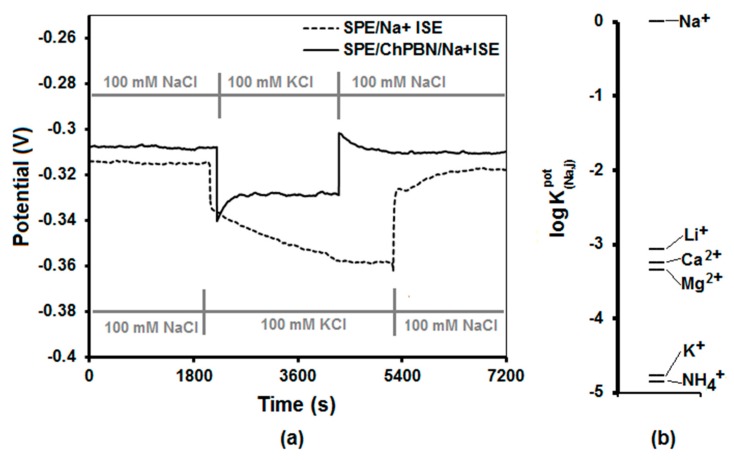
Water layer test and selectivity test of SPE/ChPBN/Na^+^ISE: (**a**) Water layer tests for the SPE/Na^+^ISE and SPE/ChPBN/Na^+^ISE in 0.1 M NaCl as the primary ion and 0.1 M KCl as the interfering ion; (**b**) potentiometric selectivity coefficients of SPE/ChPBN/Na^+^ISE. See the text for the details.

When an SS-ISE is exposed to aqueous solutions for a long period of time, a thin water layer may form between the ISE and the solid state electrode. The water layer is essentially an isolated reservoir because the equilibration of ions with respect to the surrounding environment can be delayed by the diffusion. In the presence of interfering ions, the equilibrium can be further delayed. For example, when the water layer contains K^+^ ions while the surrounding has Na^+^ ions, it takes time to equilibrate by replacing the K^+^ ions with the Na^+^. This causes a drift and delayed response of the Na^+^ISE. Based on this principle, a water layer test is devised [64]. Figure 8a shows the time-dependent potentiometric response when SPE/Na^+^ISE and SPE/ChPBN/Na^+^ISE were submerged in a series of ionic solutions: (i) the primary ion solution ([Na^+^] = 100 mM, [K^+^] = 0 mM; initially submerged for 15 days), (ii) the interfering ion (K^+^) solution ([Na^+^] = 0 mM, [K^+^] = 100 mM) and (iii) back to the primary ion solution. The ChPBN solid contact clearly improved the Na^+^ISE’s response. This indicates that the water layer formation was reduced with the presence of the ChPBN layer.

The selectivity of SPE/ChPBN/Na^+^ISE towards the primary ion (i.e., Na^+^) was examined by a separate solution method where the selectivity coefficient (${\mathrm{logK}}_{\mathrm{ij}}^{\mathrm{pot}}$) were obtained from the response potentials in the presence of different interfering ions [66,67]. The (${\mathrm{logK}}_{\mathrm{ij}}^{\mathrm{pot}}$) can be defined as the direct function of the differences of the individual potentials for the primary ion, I, and the interfering ion, j, of the same ionic activity. The alkali metal or alkali earth metal chloride solutions were used in the separate solution method keeping the same ion activity as the primary ion (Na^+^). In Figure 8b, (${\mathrm{logK}}_{\mathrm{Na},j}^{\mathrm{pot}}$) represents the selectivity coefficients of interfering ions w.r.t. the Na^+^ ion and were found to be 10^3^–10^4^-times lower in the case of interfering ions, which supports the selectivity towards the primary ion (i.e., Na^+^).

## 4. Conclusions

In this work, ChPBN was employed as a solid contact to enhance the output stability of all-solid-state Na^+^ISE. A synergy between a large redox capacitance contributed by PB and a high double layer capacitance contributed by chitosan provided significant electrochemical capacitance to the system. The high bulk capacitance contributed to the improved stability of the resulting Na^+^ISE. SPE/ChPBN/Na^+^ISEs showing good Nernstian response for sodium in a wide linear range. The highly porous structure of the ChPBN layer provided a high interfacial area against the outer layer of the PVC-based sodium selective membrane; the high interfacial area enhanced the effectiveness of the ion sensing. The selectivity was also found to be 10^3^–10^4^-times higher towards Na^+^ when tested with interfering ions. The measured sensitivity, selectivity and stability of the resulting modified Na^+^ISE confirmed that our ChPBN is a good solid contact material that can be used as an alternative choice to conducting polymers and nanostructured carbon materials in potentiometric ion-selective electrodes. As the next step, we aim to utilize the ChPBN-based potentiometric ion-selective electrode in a miniaturized biosensor for application in wearable electronics.

## Figures and Tables

**Table 1:** Capacitance and interfacial resistance obtained from Nyquist plots in Figure 4c by Boback’s protocol [11].

Electrode Surface Condition	Capacitance (µF)	Electrode/Solution Interface Resistance (Ω)
Screen-printed carbon (bare SPE)	3.44	417
SPE/chitosan	116.2	414
SPE/PB	13.1	365
SPE/ChPBN	154.5	444

**Table 2:** Capacitance and interfacial resistance values obtained by chronopotentiometry (CP) and electrochemical impedance spectroscopy (EIS) from the SPE/ChPBN/Na^+^ISE and SPE/Na^+^ISE electrodes.

Method	Parameter	SPE/Na^+^ISE	SPE/ChPBN/Na^+^ISE
CP (polarized at ±100 nA)	Potential drift (µV/s)	643 ± 4.55	288 ± 2.01
	Capacitance (µF)	457 ± 6.49	737 ± 8.51
	Membrane resistance, *R_m_* (kΩ)	372.31 ± 7.03	89.41 ± 4.71
CP (polarized at ±1 nA)	Potential drift (µV/s)	57.50 ± 2.36	12.60 ± 0.90
EIS	Capacitance (µF)	3.44 ± 0.35	13.10 ± 0.78
	Charge transfer resistance, *R_ct_* (kΩ)	88.59 ± 0.38	6.21 ± 0.20

## Supplementary Materials

The following are available online at http://www.mdpi.com/1424-8220/17/11/2536/s1. Figure S1: Current response as a function of scan rate; Table S1: Comparative representation of the analytical parameters for all-solid-state ion-selective electrode.
