# Supplementary material for: All-Solid-State Sodium-Selective Electrode with a Solid Contact of Chitosan/Prussian Blue Nanocomposite
Source: Sensors (Basel). 2017 Nov 3;17(11):2536. doi: 10.3390/s17112536 (PMC5713653; doi:10.3390/s17112536)
Supplement: Supplementary file 1 [file sensors-17-02536-s001.pdf]

SUPPORTING INFORMATION

All-Solid-State Sodium Selective Electrode with a Solid Contact of Chitosan/Prussian blue Nanocomposite

Tanushree Ghosh[1,2,3], Hyun-Joong Chung[1,*], Jana Rieger[2,3,*]

[1]Department of Chemical and Materials Engineering, Faculty of Engineering, Department of Chemical and Materials Engineering, University of Alberta, Edmonton, Alberta, Canada.

[2]Institute for Reconstructive Sciences in Medicine (iRSM), Misericordia Community Hospital, Edmonton, Alberta, Canada, Canada.

[3]Department of Communication Sciences and Disorders, Faculty of Rehabilitation Medicine, University of Alberta, Edmonton, Alberta, Canada.

* Corresponding Authors: chung.hj13@ualberta.ca (HJC) and jana.rieger@ualberta.ca (JR)

Supplementary Figure

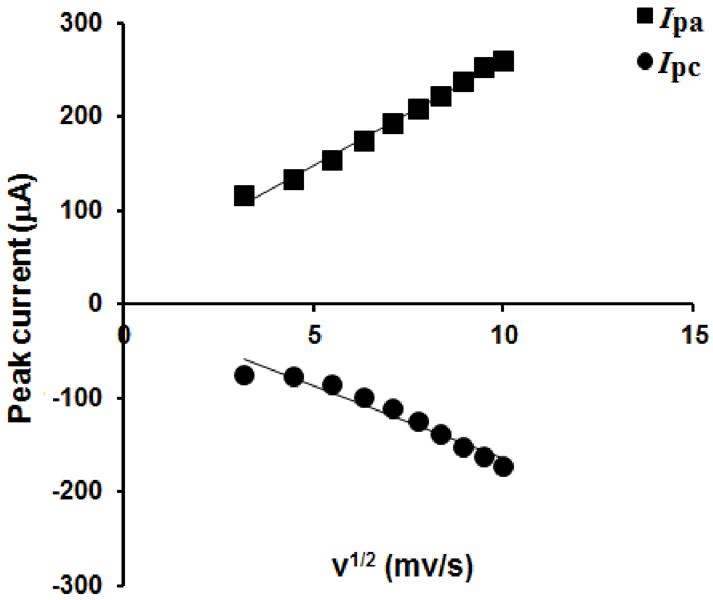

Figure S1: Current response as a function of scan rate. *I*pa and *I*pc represent anodic and cathodic peak currents, respectively.

Supplementary Table

Table S1: Comparative representation of analytical parameters for all-solid-state ion selective electrode

Working electrode	Solid contact	Target Ion	Capacitance of solid contact (μF)	Potential drift (μV/s)	Charge transfer resistance (Ω)		Slope (mV/dec)	Linear range (M)	Ref
					At solid contact/solution interface	At (solid contact + Ion selective membrane)/solution interface			
Glassy carbon disk	PEDOT(PSS)<br>PEDOT(Cl)	K^+	162<br>190	Not provided		8×10^5	Not provided	10^{-6} - 10^{-1}	[12]
Glassy carbon	Fullerene Bimodal pore C_{60}	Pb^{+2}	90	11.1		0.25×10^6	28.8	10^{-9} -10^{-3}	[19]
Glassy carbon disk	7,7,8,8-tetracyanoquinodimethane (TCNQ)	K^+<br>Na^+	132<br>154	11.1<br>9.2	333<br>425		59.24<br>58.68	$10^{-6.5}$ -10^{-1}<br>10^{-6} -10^{-1}	[32]
Platinum	Thick polypyrrole (PPy) film	Na^+	Not provided	9.23×10^{-3}		70×10^3	55.6<br>56.9	10^{-4} -10^{-1}	[8]
Platinum,	Poly(3-octylthiophene) (POT)	Li^+<br>Ca^{2+}<br>Cl^-	Not provided	Not provided		4.1×10^6<br>6.1×10^6<br>0.14×10^6	56.0<br>30.0<br>-62.6	Not provided	[9]
Glassy	polyaniline	Li^+	Not provided	9.23×10^{-3}	Not		57.8	10^{-3} -10^{-1}	[10]

carbon	doped poly(3-octylthiophene)	Ca^{2+}		5.78×10^{-3}	provided		27.0	10^{-5} -10^{-1}	
Gold	Prussian blue	K^{+}	Not provided	Not provided	Not provided		42.0	10^{-6} -10^{-1}	[29]
Nickel mesh	three-dimensionally ordered macroporous (3DOM) carbon	K^{+}	Not provided	3.25×10^{-3}	Not provided		59.2	10^{-6} -10^{-3}	[16]
Copper	single-walled carbon nanotubes (SWCNTs)	K^{+}	59	85		13.5×10^{6}	58.1	10^{-5} -10^{-3}	[17]
Glassy carbon	Fullerene (C_{60})	K^{+}	1.26×10^{-6}	Not provided	1728		55	10^{-4} -10^{-2}	[18]
Glassy carbon	Reduced graphene oxide	K^{+}	78.1	12.8		8.32×10^{6}	58.4	$10^{-5.8}$ -10^{-1}	[20]
Glassy carbon	Reduced graphene oxide	Ca^{2+}	Not provided	2.77×10^{-3}	75.6		29.5	10^{-5} -$10^{-2.5}$	[21]
Glassy carbon	Graphene sheet	K^{+}	91	55		2.4×10^{6}	59.2	$10^{-4.5}$ -10^{-1}	[22]
Glassy	Carbon Black	K^{+}	51	4.19×10^{-3}	Not		59.1	$10^{-6.5}$ -10^{-1}	[64]

carbon disk					provided				
Arenethiolate monolayer-protected Au clusters	tetrakis(4-chlorophenyl)borate (TB−) anion doped nanocluster films	K^+	117.6	8.5		4.52×10^{6}	58.8	10^{-6} -10^{-1}	[66]
Gold disk	colloid-imprinted mesoporous (CIM) carbon	K^+	1×10^{3}	3.61×10^{-4}		360×10^{3}	59.5	$10^{-5.2}$ -10^{-1}	[24]
Porous carbon (PC) electrodes	Porous carbon (PC)	K^+	Not provided	212		12.75×10^{6}	57.9	10^{-5} -10^{-2}	[23]
Microfabricated 30 nm Cr/50 nm Au	poly(3,4-ethylenedioxythiophene) polystyrene sulfonate (PEDOT:PSS)	Na+<br>K+	Not provided	Not provided	Not provided		64.2<br>61.3	10^{-3}–160×10^{-3}<br>10^{-3}–32×10^{-3}	[36]
Carbon-Screen printed	Chitosan-PB nanocomposite	Na^+	154	3.61×10^{-4}		6.21×10^{3}	52.4	10^{-4} -1	Present work

electrode
